# Obturator neuropathy from SLE lymphadenopathy: an interdisciplinary diagnostic challenge

**DOI:** 10.1007/s10067-026-08009-x

**Published:** 2026-03-03

**Authors:** Ulf Krister Hofmann, Jörg Henes, Julius Michael Wolfgart, Gabriele Reiff, Frank Traub

**Affiliations:** 1 Department of Orthopaedic, Trauma, and Reconstructive Surgery, University Hospital RWTH Aachen, Aachen, Germany; 2 Centre for Interdisciplinary Clinical Immunology, Rheumatology and Autoinflammatory Diseases, University Hospital Tübingen, Tübingen, Germany; 3 Department of Internal Medicine II, University Hospital Tübingen, Tübingen, Germany; 4 Department of Internal Medicine I, Hospital Freudenstadt, Freudenstadt, Germany; 5 Department of Orthopaedics and Traumatology, University Medical Center of the Johannes Gutenberg University Mainz, Mainz, Germany

**Keywords:** Lymphadenopathy, Nerve compression, Obturator nerve, Systemic lupus erythematosus

## Abstract

**Objective:**

To describe an unusual cause of thigh myopathy and nerve compression in a patient with systemic lupus erythematosus (SLE), illustrating the importance of interdisciplinary collaboration.

**Case presentation:**

A 26-year-old woman with a 10-year history of SLE presented with new-onset right thigh pain and restricted hip movement. Knee pathology was excluded, and symptoms were initially attributed to hydroxychloroquine treatment. Despite conservative management, pain worsened. MRI revealed multifocal myositis in the gluteal and adductor regions with signal changes in the sciatic nerve and enlarged groin lymph nodes. Repeated histopathological, neurological, and radiological examinations remained inconclusive until a pelvic MRI showed bilateral lymphadenopathy along the lumbar plexus, compressing the obturator nerve.

**Results:**

Treatment with high-dose prednisolone led to rapid clinical improvement with resolution of pain and restoration of hip mobility, accompanied by radiological resolution of muscular and nodal changes.

**Conclusion:**

This case demonstrates an uncommon manifestation of active SLE with pelvic lymphadenopathy causing obturator nerve compression and secondary thigh myopathy. Early interdisciplinary evaluation was essential for diagnosis and management in this complex autoimmune presentation.

## Introduction

A young woman aged 26 years with known systemic lupus erythematosus (SLE) presented to our hospital with new pain in her right thigh and restriction of hip movement. Obturator neuropathy due to lupus-related lymphadenopathy is a rare manifestation of SLE. Awareness of this mechanism may help clinicians avoid misinterpretation as primary myositis and prompt timely interdisciplinary evaluation.

## Medical history

SLE was first diagnosed in 2004 at the age of 15, when the patient presented with scattered discoid cutaneous lesions, arthritis, photosensitivity, a malar rash, and lingual mucosal erosions. Residual scarring from the discoid lesions was present in later examinations, whereas associated alopecia was not explicitly documented. Overall, this presentation is consistent with overlapping cutaneous lupus phenotypes rather than a single distinct subtype. Laboratory results were initially positive for antinuclear antibodies and anti-double-stranded DNA but negative for rheumatoid factor and anti-citrullinated protein, cardiolipin, and beta-2 glycoprotein antibodies. However, histopathological skin biopsy analysis, in conjunction with the clinical presentation, confirmed SLE.

An initially successful course of hydroxychloroquine was discontinued after 2 years because of reduced efficacy and drug intolerance manifested by persisted nausea. Intermittent systemic prednisolone regimes followed with local hydrocortisone ointment and celecoxib intake on demand. After the disease was exacerbated as a result of intensified sun exposure during the patient’s vacation in the Mediterranean, methotrexate treatment was started in 2008. In 2010, cortisone therapy was briefly administered in response to a relapse of the cutaneous and arthritic manifestations. Methotrexate treatment was thereafter augmented with hydroxychloroquine, and the patient showed good clinical remission with no symptomatic arthritis and cutaneous exacerbations. Methotrexate was initiated given ultrasound evidence of active synovitis, slightly reduced C3 levels, and intermittent cutaneous manifestations. Throughout these years, the patient took about 1000 IU of vitamin D per day.

At the beginning of 2015, cervical lymphadenopathy with a maximum lymph node size of 2 cm was observed, but the patient did not agree to their removal for diagnostic purposes. For the first time, she reported right knee pain that progressed to tenderness on palpation and muscular tension during movement of the right thigh. A primary pathology in the knee having been excluded by the results of magnetic resonance imaging (MRI) and the intermittent arthritic history, these symptoms were attributed to hydroxychloroquine-induced myopathy [1]; the therapeutic strategy was maintained with the addition of physiotherapy of the right leg.

## Current problem

**Fig. 1:**
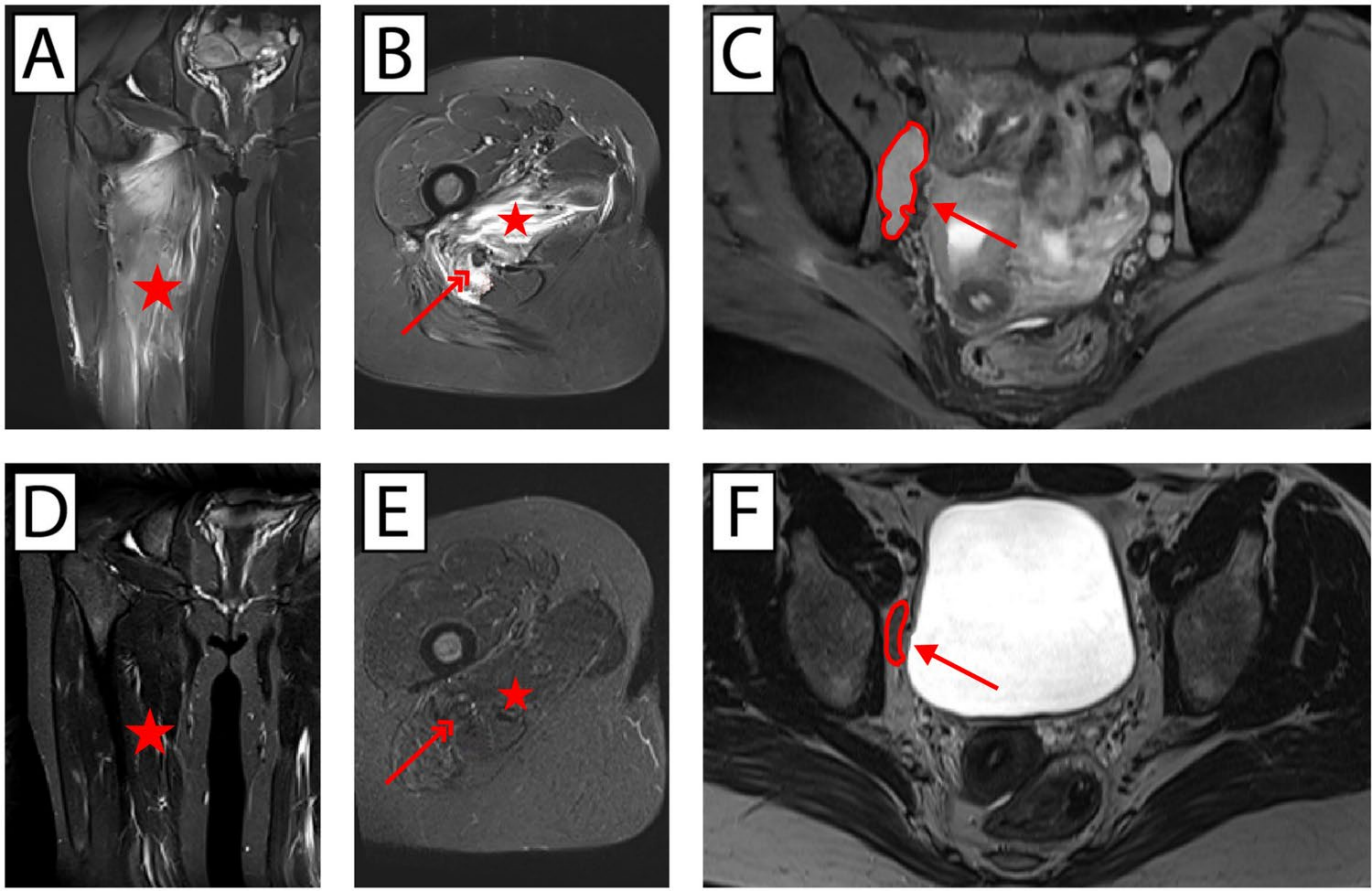
Magnetic resonance imaging at presentation (**A**–**C**) and after successful treatment (**D**–**F**). **A**,** D** Coronal T2 TIRM (short tau inversion recovery) and (**B**,** E**) axial T2 TIRM image of the right thigh to illustrate edema formation in the muscle by the hyperintensive signal. **C** Axial fast recovery FSE PD (proton density) with fat saturation and (**F**) T2 TSE (turbo spin echo) image of the pelvis to visualize size and shape of the lymph nodes. The red asterisk marks the adductor magnus muscle, the red double arrow the sciatic nerve. The single arrow depicts the obturator nerve; the enlarged lymph node is encircled by a red line. It can be observed that after successful treatment, the signal for edema has vanished from the adductor muscle and the enlarged lymph node has strongly diminished in size

**Fig. 2:**
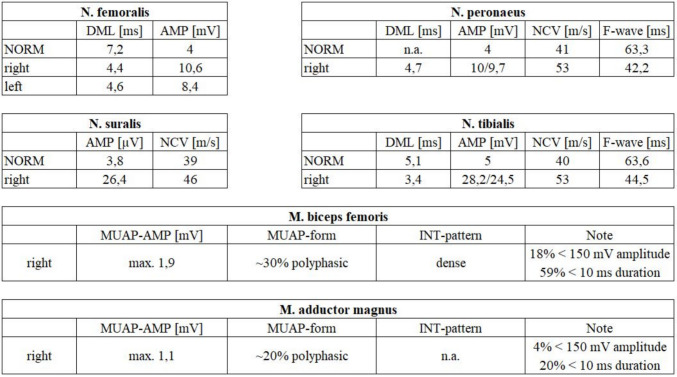
Electroneurography on the 21 st of April 2016. Nerve conduction studies showed no relevant abnormalities, while electromyography demonstrated only mild, non-specific myopathic changes, highlighting the limited sensitivity of standard testing for mild obturator nerve involvement. Diagram shows motor nerve conduction study of the nervus femoralis on both sides, nervus peroneus on the right side, nervus tibialis on the right side, and sensory nerve conduction study of the nervus suralis on the right side. In addition, electromyography of the musculus biceps femoris and musculus adductor magnus of the right side. Abbreviations: NORM—reference value, DML—distal motor latency, AMP—amplitude, NCV—nerve conduction velocity, MUAP—motor unit action potential-amplitude, INT—interference

One year later (beginning of 2016), the patient presented for follow-up and described increasing symptoms in her right leg without, however, any sensorimotor deficit. An MRI of the right leg performed and interpreted outside our department of radiology was suggestive of myositis ossificans. A repeat of this imaging in our department showed aspects of a multifocal myositis in the gluteal region, around the hip joint and along the adductor magnus muscle on the right side. Moreover, signal alterations of the sciatic nerve were described, as well as accentuated lymph nodes in the groin region. While no relevant changes in the size of the lymph nodes were detected in comparison to those from the previous MRI from November 2015, signal changes in the muscles appeared to increase (Fig. 1A–C). To clarify the etiology, we performed a biopsy of the altered muscle tissue in February 2016. However, except for a slightly increased endomysium and partially upregulated major histocompatibility complex type 1, no pathological changes were found. Because symptoms further increased, a re-biopsy was performed in March 2016. This time, histopathological analyses showed a lack of adenosine monophosphate deaminase and type I muscle fiber dominance. No neuromuscular pathological alterations were detected. Electroneurography in our department of neurology from April 2016 showed no pathological condition of the femoral, tibial, sural or peroneal nerve but did indicate myopathic changes of the biceps femoris muscle. Electromyography showed no evidence of myositis but did reveal myopathic changes in the right biceps femoris muscle. Neurological evaluation concluded that no indications were present for an effect on the obturator nerve (Fig. 2).

In a new MRI in April 2016 where, for the first time, the pelvic region was also included in the imaging protocol, a notable bilateral increase in the number and size of suspicious lymph nodes in the iliac and groin region was present. In addition, enlarged lymph nodes were detected along the lumbar plexus on both sides. The signal alterations in the thigh muscles and the gluteal area remained unchanged, as did the changes in the sciatic nerve.

Interestingly, the obturator nerve on the right side appeared to be compressed by an enlarged lymph node (Fig. 1C). Subsequent whole-body computed tomography (CT) imaging that included a CT image of the skull showed generalized lymphadenopathy with enlarged lymph nodes bilaterally in the cervical, nuchal, supraclavicular, infraclavicular, retroperitoneal, and pelvic regions. To rule out the possibility of lymphoma, we performed a biopsy of a lymph node from the right pelvis in April 2016. Results showed reactive changes in the node architecture, including polyclonal plasmacytosis, and a positive result for Epstein–Barr virus (EBV) in some scattered cells, but no hint of malignant transformation. Thorough serological screening showed no viral infections for human parainfluenza, measles, mumps, rubella, tick-borne encephalitis, herpes simplex virus 1/2, varicella zoster virus (VZV), cytomegalovirus, Epstein–Barr virus (a positive result for Epstein–Barr nuclear antigen-IgG being indicative of past infectious mononucleosis), human herpesvirus 6, human immunodeficiency virus, or hepatitis A/B/C and no serological hint of *Borrelia burgdorferi*, *Treponema pallidum*, or *Mycobacterium tuberculosis*.

As a result of these examinations, we concluded that lymphadenopathy was part of the SLE. A prednisolone regimen was thus initiated in May 2016 starting at 150 mg per day with a subsequent rapid reduction in dosage. It was administered orally, as the patient was clinically stable and did not exhibit life-threatening organ involvement requiring intravenous pulse therapy. This led to rapid clinical and radiological improvement of the symptoms in the right leg and pelvic lymph nodes (Fig. 1D, E). This clinical response was interpreted as proof that SLE was the underlying disease causing the lymphadenopathy and explained the muscular complaint and MRI changes.

Because of the problematic course of the disease over the past months, it was recommended that the patient’s treatment temporarily be augmented with azathioprine in addition to methotrexate before continuing with azathioprine without methotrexate as a steroid-sparing strategy due to the complex disease course. This temporary augmentation in anti-rheumatic treatment was accompanied by prophylactic administration of famciclovir, which was indicated because of a severe VZV infection characterized by localized vesicular eruption in the C5 and C6 dermatome and subsequent left-sided radiculopathy with muscle strength of 1/5 for external rotation and abduction of the shoulder and elbow flexion. The diagnosis was confirmed by cerebrospinal fluid analysis, which demonstrated pleocytosis (155 cells/µL) and a positive VZV PCR result. In addition, a new Horner’s syndrome on the left side had appeared that was attributed to nerve compression due to VZV-associated lymphadenopathy visible on MRI. Treatment with intravenous acyclovir and then oral valaciclovir was initiated, accompanied by pregabalin for neuropathic pain. By October 2016, Horner’s syndrome had again disappeared, and the sensorimotor deficits of the left arm were slowly recovering.

**Fig. 3:**
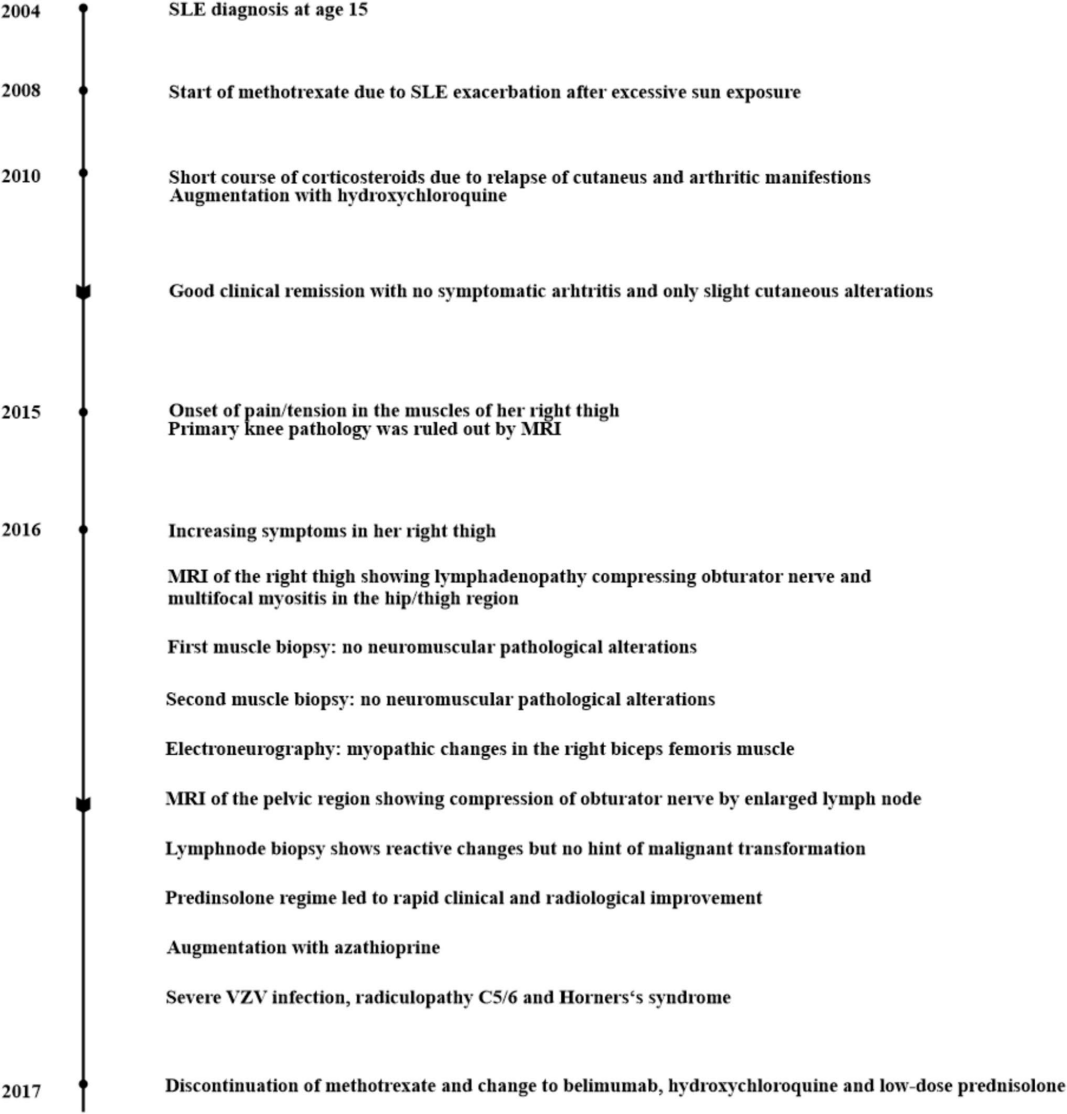
Main events on the course of the case report. Abbreviations: SLE—systemic lupus erythematosus, MRI—magnetic resonance imaging, VZV—varicella zoster virus

Since then, inflammatory parameters have been well controlled under azathioprine therapy accompanied by famciclovir prophylaxis. Pregabalin intake was discontinued, and neuropathic pain is no longer present. The right leg has completely recovered with no residual deficits of Horner’s syndrome. The patient is still building up muscle strength of the left arm, which remains, to date, reduced in function with muscle power of 2/5 for external rotation and abduction of the shoulder and 4/5 for elbow flexion. By February 2017, methotrexate was also discontinued with a clinically stable presentation and no elevated inflammatory parameters. Treatment was transitioned to belimumab for long-term disease control, accompanied by hydroxychloroquine 200 mg once daily and prednisolone 5 mg once daily. Belimumab was selected as maintenance therapy as part of a standard B cell-targeted treatment strategy for systemic lupus erythematosus, aiming to sustain remission and minimize long-term corticosteroid exposure; this decision was based on overall disease control rather than a specific indication for lymphadenopathy and reflected its established efficacy and safety profile in non–organ-threatening SLE. The patient has no residual symptoms, and the disease has been well controlled and stable with no increase in serum inflammatory parameters (for timeline and main events, see Fig. 3).

## Differential diagnosis

The differential diagnosis included systemic lupus erythematosus–associated myositis, steroid-induced myopathy, hydroxychloroquine-related myopathy, overlap myositis, infection, and malignancy. Steroid myopathy was considered unlikely due to the focal nature of symptoms, the presence of regional MRI signal changes, and the rapid improvement following escalation rather than reduction of glucocorticoid therapy. Hydroxychloroquine-related myopathy was initially suspected; however, the asymmetric presentation, lack of generalized progressive weakness, and absence of characteristic histopathological findings on repeated muscle biopsies argued against this diagnosis. An overlap myositis was considered unlikely in the absence of typical clinical features and definitive histological evidence. Myositis ossificans was an early outside misread that was ultimately ruled out by muscle biopsy.

Importantly, the myositis-like MRI signal abnormalities are best explained as denervation-related muscle edema secondary to obturator nerve compression caused by lupus-associated lymphadenopathy rather than primary inflammatory myositis. Extensive imaging and lymph node histology did not support infection or malignancy. The rapid clinical and radiological response to immunosuppressive treatment ultimately supported active SLE with secondary neuromuscular involvement.

## Discussion

SLE is an autoimmune disease presenting with a wide variety of symptoms. Although inflammatory joint pain and skin manifestations are common, lymphadenopathy, myositis, and neuropathy can also be observed. Lymphadenopathy has been described in about 12–80% of all cases of SLE and appears to be an indicator for disease activity [2–4]. Histologically, lymph nodes present non-specific follicular hyperplasia, necrotizing lesions, and hematoxylin bodies and can imitate Castleman’s disease [5]. Reactive lymphoproliferation mimicking Epstein–Barr virus–associated disease represents another diagnostic pitfall. SLE-associated lymphadenopathy typically responds well to corticosteroids and immunosuppressive therapy, as observed in the present case, in contrast to lymphoproliferative malignancies, which generally require oncologic treatment. Skeletal muscle involvement has been described in 4–16% of patients [6] and usually presents in the form of muscle weakness, myalgia, and atrophy [7]. Histological characteristics of these myopathic muscles are perivascular/perimysial inflammation, type 1 muscle fiber predominance, and type 2 fiber atrophy [6]. Peripheral neuropathy apparently has a comparable incidence in SLE ranging from 2 to 18% [8] and can be classified into different subtypes, such as axonal, small-fiber, or demyelinating neuropathies [9]. While these phenomena are usually analyzed in the literature with a focus on their direct link with inflammatory SLE, our case report offers an interesting causality chain: Pelvic lymphadenopathy led to obturator neuropathy, which eventually created the myopathic image of the adductor region. Obturator neuropathy can be diagnosed based on medial thigh pain, weakness in thigh adduction, sensory loss over the medial thigh, and confirmed by EMG findings in the adductor muscles. The initially normal electroneurography findings are likely attributable to timing-related limitations and for that time being a mild degree of obturator nerve compression, which may have been insufficient to produce detectable abnormalities on standard neurophysiological testing. It should be noted that denervated muscle may occasionally exhibit “pseudomyopathic” electromyographic features, such as early recruitment or low-amplitude motor unit action potentials (MUAPs), particularly in the early stages of denervation or during reinnervation, which may account for the apparent discrepancy between the clinical diagnosis and the electrodiagnostic findings. While MRI excels at detecting inflammation (T2-hyperintensity), this sensitivity can also be misleading, as T2-weighted images may aggravate findings. In retrospect, the diagnosis is apparent, but initially, no single finding was pathognomonic: Electroneuronography/electromyography did not record a clearly pathological pattern, and histology did not offer a conclusive result. In addition, the initial MRI, which focused on the symptomatic thigh region, did not present a plausible explanation but simply described the present myositis. Ultimately, the imaging, histopathological, and neurophysiological finding changes were most consistent with denervation-related muscle edema secondary to obturator nerve compression, rather than primary inflammatory myositis suggesting that the observed MRI findings showed “myositis-like” changes. This interpretation is supported by inconclusive muscle biopsies, the limitations of neurophysiological testing, and the rapid resolution of muscular edema following immunosuppressive treatment targeting the underlying SLE-associated lymphadenopathy. The explanation of the originally reported symptom of tenderness and reduced flexibility of the thigh can be explained as the cumulative effect of different members of a chain, each contributing to the final clinical presentation. Diagnosis required multiple interdisciplinary meetings involving rheumatology, pathology, neurology, radiology, and orthopedic specialists. Each contributed essential perspectives that were impossible from any single specialty. This underlines the never-ending necessity for intensive interdisciplinary collaboration when dealing with such a complex disease as SLE.

## Limitations

This report is limited by its single-patient design and the retrospective interpretation of findings. Nevertheless, the consistent clinical, radiological, and therapeutic response supports the proposed pathophysiological mechanism.

## Data Availability

Not applicable.

## Abbreviations

CT: Computed tomography
EBV: Epstein–Barr virus
MRI: Magnetic resonance imaging
SLE: Systemic lupus erythematosus
VZV: Varicella zoster virus
